# Transmissibility and Curability of Cancer

**Published:** 1907-08

**Authors:** William Seaman Bainbridge

**Affiliations:** New York


### Transmissibility and Curability of Cancer.

By WILLIAM SEAMAN BAINBRIDGE, New York.

[*Author's Abstract.*]

DR. William Seaman Bainbridge of New York City calls attention to the growing fear of cancer on the part of people of all classes. He attributes this to the theories of heredity, congenital transmission, and infectiousness or contagiousness as causal factors in the production of the disease. The fear of the contagiousness of cancer has been aroused by the exploitation of the subject in the public press. After reviewing the evidence *pro* and *con* of these theories, he calls attention to the following points, adduced from the mass of conflicting evidence, which, pending the solution of the "cancer problem," will lead no one into danger: (1) That the hereditary and congenital acquirement of cancer are subjects which require much more study before any definite conclusions can be formulated concerning them. (2) That in the light of our present knowledge they hold no special element of alarm. (3) That the contagiousness or infectiousness of cancer is far from proved. (4) That evidence to support the theory of contagion or infection is so incomplete and inconclusive that the public need not concern itself with it. (5) That the public need merely be instructed to apply the same precautionary measures as should be brought to bear in the care of any ulcer or open wound. (6) That the danger of the accidental acquirement of cancer is far less than from typhoid fever, syphilis or tuberculosis. (7) That in the care of cancer cases there is much more danger to the attendant of sceptic infection, of blood poisoning from pus organisms, than from any possible acquirement of cancer. (8) That the communication of cancer from man to man is so rare, if it really occurs at all, that it can practically be disregarded. (9) That in cancer, as in all other disease, attention to diet, exercise, and proper hygienic surroundings, is of the utmost importance. (10) That cancer is local in its beginning. (11) That, when accessible, it may, in its incipiency, be removed by radical operation so perfectly that the chances are overwhelmingly in favor of its non-recurrence. (12) That once it has advanced beyond the stage of cure, in many cases suffering may be palliated and life prolonged by radical surgical means. (13) That while other methods of treatment may, in some cases, offer hope for the cancer victim, the evidence is conclusive that surgery, for operable cases, affords the surest means of cure.

